# Correlation of surgical volume in gynecological cancer centers with the quality of ovarian cancer care

**DOI:** 10.1007/s00432-025-06288-6

**Published:** 2025-08-28

**Authors:** Olaf Ortmann, Rebecca Roth, Birgit Klages, Christoph Kowalski, Johannes Bruns, Pauline Wimberger, Jalid Sehouli, Matthias W. Beckmann, Susanne Schüler-Toprak

**Affiliations:** 1 University Medical Center Regensburg, Comprehensive Cancer Center (CCC) Würzburg, Erlangen, Regensburg, Augsburg (WERA), National Center for Tumor Diseases (NCT), Department of Gynecology and Obstetrics, Landshuter Str. 65, Regensburg, Germany; 2 Department for Health Services Research, German Cancer Society, Kuno-Fischer- Strasse 8, 14057 Berlin, Germany; 3 Department for Certification, German Cancer Society, Kuno-Fischer-Strasse 8, 14057 Berlin, Germany; 4 German Cancer Society, Kuno-Fischer-Strasse 8, 14057 Berlin, Germany; 5 Department of Gynecology and Obstetrics, Medical Faculty and University Hospital Carl Gustav Carus, CCC, NCT Dresden, Technische Universität Dresden, Dresden, Germany; 6 Department of Gynecology with Center for Oncological Surgery, Campus Virchow Klinikum, Charité-Universitätsmedizin Berlin, CCC, NCT, Corporate Member of Freie Universität Berlin, Humboldt-Universität zu Berlin and Berlin Institute of Health, Berlin, Germany; 7 Department of Gynecology and Obstetrics, University Hospital, Friedrich-Alexander-Universität Erlangen – Nürnberg (FAU), CCC Erlangen-EMN, CCC WERA, Bavarian Cancer Research Center (BZKF), Erlangen, Germany

**Keywords:** Ovarian cancer, Surgical volume, Complete surgical staging, Macroscopic complete resection

## Abstract

**Purpose:**

Surgical volume in ovarian cancer treatment has been discussed to influence survival. Completeness of staging in early and macroscopic complete resection in advanced ovarian cancer are indicators of treatment quality and surrogate parameters for outcome. This study examines their association with case volume in certified gynecological cancer centers.

**Methods:**

Certification audit data from ovarian cancer cases treated in 193 gynecological cancer centers certified by the German Cancer Society between 2020 and 2022 were analyzed. In our study 3,881 patients with FIGO stage I-IIIA and 7,219 patients with FIGO stage IIB-IV ovarian cancer were included. Case volume thresholds of 10, 15, 20 and 25 annual cases per hospital were explored for both quality indicators.

**Results:**

Higher-volume centers consistently performed complete surgical staging in FIGO stage I-IIIA ovarian cancer patients more frequently. For example, centers treating < 15 cases annually had a lower likelihood of complete staging (OR 0.82; 95% CI 0.69–0.98; *p* = 0.031). Macroscopic complete resection was achieved in 72.32–75.64% of FIGO stage IIB-IV ovarian cancer cases across all four thresholds. High-volume centers performed macroscopic complete resection about 2 percentage points more often.

**Conclusion:**

These data indicate that case volume of cancer centers is not necessarily an indicator for quality of treatment. Since certification audit data does not account for primary surgery or neoadjuvant chemotherapy, the inherent bias caused by differences in resection rates between these two treatment strategies must be acknowledged.

## Purpose

In Germany, approximately 7,000 cases of ovarian cancer are diagnosed every year. It is the second most frequent gynecological cancer and the leading cause of death from gynecological cancer (Robert Koch-Institut 2023). Ovarian cancer care is characterized by complex multidisciplinary interventions. Substantial progress has been achieved in recent years especially in the field of medical treatments. A number of studies have demonstrated the importance of appropriate surgery of ovarian cancer in early and advanced stages of the disease (Du Bois et al. 2009; Wimberger et al. 2023). The majority of ovarian cancer cases is diagnosed in advanced stages. It has been clearly demonstrated that optimal cytoreductive surgery (no residual disease) has a strong effect on survival while lymphonodectomy in the absence of bulky nodes does not (Harter et al. 2019). It was also recognized that treatment in specialized cancer centers may lead to improvement of outcome (Vernooij et al. 2009; Bristow et al. 2015; Cliby et al. 2015; Carter et al. 2018; Nasioudis et al. 2019; Knisely et al. 2020; Machida et al. 2022; Millert-Kalińska et al. 2022; Schmitt et al. 2023).

However, it is less clear which factors determine improved outcomes in routine care. Based on previous studies, there is consensus that guideline concordant treatment in qualified cancer centers is a major factor that leads to better survival rates. Since cytoreductive surgery performed in advanced stages of the disease is a very complex procedure often requiring multidisciplinary expertise, it was hypothesized and shown in some studies that case volume is an important factor which determines survival. However, this is a matter of controversy for several reasons. Previous studies have proposed different definitions for low, moderate and high-volume centers. For example, Bristow et al. have examined survival of ovarian cancer in National Cancer Institute Comprehensive Cancer Centers (NCI-CCC), non NCI-high volume hospitals (≥ 10 cases/year), or low volume hospitals (< 10 cases/year) (Bristow et al. 2015). Although NCI-CCC and non-NCI-high volume hospital treatment volumes were similar (14.5 vs. 14.6 cases/year), adherence to NCCN guidelines was poorer in non-NCI-high volume hospitals (OR 0.83; 95% CI 0.70–0.99) an in low volume hospitals (OR 0.56; 95% CI 0.47–0.67) than in NCI-CCC. This was associated with better survival of patients treated in NCI-CCC (77.9 months) compared to non-NCI high volume hospitals (51.9 months) and low volume hospitals (43.4 months). Another study examined the patterns of ovarian cancer care in the United States (US) using data from the National Cancer Data Base (NCDB) capturing 80% of all US cases: high volume centers were less likely to deliver non guideline care (Cliby et al. 2015). Not receiving NCCN guideline-concordant care was associated with worse overall survival (HR 1.40; 95% CI, 1.36–1.45) whereas overall survval was best in highest volume centers (> 29 cases, HR 0.91; 95% CI 0.81–0.96). Thus, guideline concrdant treatment had a larger effect than hospital volume.

Given the controversy on volume and other factors that may determine outcome we examined the association of volume with quality of surgery for ovarian cancer patients in gynecological cancer centers certified by the German Cancer Society (Deutsche Krebsgesellschaft, DKG). These specialized cancer centers have been established since 2007. Certified centers undergo yearly onsite clinical audits organized by an independent agency (OnkoZert) carried out by specially trained oncological experts and have to fulfill numerous requirements set out by a multidisciplinary expert group (“Certification Committee”) including patient representatives under the roof of the German Cancer Society. This committee defines and develops the requirements specification for the certification of the respective centers based on evidence-based clinical guidelines of the highest level of quality (“S3-level”), supplemented by e.g. structural requirements like staffing or technical infrastructure (Kowalski et al. 2017).

Details on the development of the requirements, the auditing, the awarding of the certificates as well as data collection and reporting have been reported previously (Kowalski et al. 2017).

Among others, the requirements for gynecological cancer centers include: the provision of care by multidisciplinary teams including at least two gynecological oncologists, multivisceral surgeons, medical oncologists as well as weekly interdisciplinary tumor conferences, and an established network providing all components of the diagnostic and treatment pathway. The required annual volume for these centers is at least 50 primary cases and a total of 75 cases of gynecological malignancies including invasive cancers and borderline ovarian tumors but and no precancerous lesions. However, these numbers are not further specified for each of the five types of gynaecological cancer (ovarian, cervical, endometrial, vulvar, vaginal cancer), leading to specialization for specific cancers in some centers and to substantial variation in the number of ovarian cancer cases across centers.

To investigate whether there is an association between case number and surgical performance quality for ovarian cancers within certified centers, we analyzed data on the two quality indicators related to ovarian cancer surgery required for certification in patients treated between 2020 and 2022: the quality of staging surgery in FIGO stage I-IIIA and macroscopic complete resection in FIGO stage IIB-IV ovarian cancer. In addition to case numbers, associations between teaching status and quality indicator results were investigated.

## Materials and methods

### Data collection

Data analyzed in this study have been obtained from the gynecological cancer centers certified by the DKG in Germany, Switzerland and Austria. To be awarded the certificate, centers must report annually center-aggregated characteristics of gynecological cancer patients in terms of quality indictors based on TNM or FIGO-classification, number of cases receiving surgery or medical treatment including neoadjuvant therapy. Among various requirements, defined quality indicators from treatment guidelines are used as key figures and minimum quantities are required for surgical expertise and other therapeutic or diagnostic procedures in order to make quality and guideline adherence measurable and verifiable and at the same time to address the quality of the process and results (Kowalski et al. 2017). For this study, we have investigated the two indicators relevant to ovarian cancer (including cancer of the fallopian tubes and peritoneum) for the three most recent data years (2020–2022) at the time of writing this report:

1. “Surgical staging of early ovarian cancer“ with the denominator “primary cases of ovarian cancer FIGO stage I-IIIA receiving surgery” and the numerator “cases of the denominator with surgical staging including laparotomy, peritoneal cytology, peritoneal biopsies, bilateral adnectomy, hysterectomy, at least infracolic omentectomy, bilateral pelvic and paraaortal lymphonodectomy”.
  and.
2. “Macroscopic complete resection in cases with advanced ovarian cancer, with the denominator “primary cases with an ovarian cancer FIGO IIB-IV receiving surgery” and the numerator “cases from the denominator with macroscopic complete resection”.
  Based on the available certification data, it is not possible to differentiate between patients who underwent primary cytoreductive surgery and those who reveiced neoadjuvant chemotherapy with subsequent surgical treatment.

### Statistical analysis

Patients were grouped according to tumor stage and treatment in centers below or above case number thresholds. Four different thresholds (10, 15, 20, 25 annual cases per hospital for the respective indicator) were explored for both indicators to differentiate between low and high volume centers. The grouping according to case number was performed on an annual basis, i.e. patients treated in a specific hospital can be in the below-threshold group in one year, while patients from the same hospital in the subsequent year may be in the above threshold group. Frequencies of patients in the denominator were pooled across years according to the threshold groups. To test for statistically significant differences, chi-square tests were calculated and corresponding unadjusted odds ratios (OR) along with 95% confidence intervals (CI) were derived. No adjustments were made due to the center-aggregated nature of the data (Kowalski et al. 2015). Centers were included in the analyses independent of the number of years they held their certificate as long as they reported data for at least one full calendar year. P-values < 0.05 were considered statistically significant. However, due to multiple tests performed and the explorative nature of the analyses p-values are only interpreted descriptively (hypotheses generation).

## Results

### Characteristics of included certified gynecological cancer centers

In the years 2020 to 2022, 20,824 ovarian cancer cases were treated in 193 centers certified for at least one of the considered years. Among the 20,824 ovarian cancer cases treated between 2020 and 2022 only those with primary ovarian cancer that received surgical treatment were included in our analyses. Finally, 3,881 (18.64%) surgical primary FIGO stage I-IIIA ovarian cancer cases and 7,219 (34.67%) surgical primary FIGO stage IIB-IV ovarian cancer cases were analyzed in this study, respectively.

Up to 77.8% of all centers that treated FIGO stage I-IIIA ovarian cancer during the observation period performed surgery on less than 10 patients in FIGO stages I-IIA per year. Only 1.1% (*n* = 3) treated > 25 patients in these stages per year (Table 1). As far as the treatment of FIGO stage IIB-IV ovarian cancer is concerned, up to 49.2% of centers treated less than 10 patients with FIGO stage IIB-IV ovarian cancer surgically per year in 2020-22. Between 10.2 and 7.3% performed surgical treatment on more than 25 FIGO stage IIB-IV ovarian cancers annually (Table 1).

**Table 1:** Number of centers treating < 10, <15, < 20 and < 25 patients with FIGO I-IIIA and FIGO IIB-IV ovarian cancer per year

	Number of centers (with evaluable data) treating:			
	< 10 patients	< 15 patients	< 20 patients	< 25 patients
FIGO I-IIIA				
2020	130/167 (77.84%)	150/167 (89.82%)	163/167 (97.60%)	164/167 (98.20%)
2021	132/175 (75.43%)	161/175 (92.00%)	172/175 (98.29%)	172/175 (98.29%)
2022	139/185 (75.14%)	171/185 (92.43%)	182/185 (98.38%)	183/185 (98.92%)
FIGO IIB-IV				
2020	71/168 (42.26%)	127/168 (75.60%)	141/168 (83.93%)	153/168 (91.07%)
2021	79/177 (44.63%)	137/177 (77.40%)	150/177 (84.75%)	164/177 (92.66%)
2022	92/187 (49.20%)	135/187 (72.19%)	153/187 (81.82%)	168/187 (89.84%)

Of the 193 included centers, 46 (23.8%) were university hospitals, 132 centers (68.4%) were non-university teaching hospitals and 15 centers (7.8%) were non-teaching hospitals.

### Surgical staging in cases of FIGO stage I-IIIA ovarian cancer

#### Frequency of complete surgical staging of FIGO stage I-IIIA ovarian cancer depending on the number of treated cases

3,881 patients with FIGO stage I-IIIA ovarian cancer treated between 2020 and 2022 were included in the analysis. Patients treated in higher volume centers received complete surgical staging more frequently. For centers treating less than 15 surgical primary ovarian cancer cases annually, the chance of complete surgical staging was reduced by 18% compared to centers with 15 or more cases (OR 0.82; 95% CI 0.69–0.98; *p* = 0.03096) (Table 2). Higher thresholds for case numbers led to bigger differences in the frequency of surgical staging between lower and higher volume centers (*p* < 0.00001).

**Table 2:** Surgical staging of FIGO stage I-IIIA ovarian cancer

Threshold	Frequency of surgical staging in centers < threshold	Frequency of surgical staging in centers ≥ threshold	OR (95% CI)	*p*-value
< 10	75.35% (1513/2008)	76.29% (1429/1873)	0.95 (0.82–1.10)	0.51556
< 15	74.94% (2192/2925)	78.45% (750/956)	0.82 (0.69–0.98)	0.03096*
< 20	74.75% (2620/3505)	85.64% (322/376)	0.50 (0.36–0.67)	< 0.00001*
< 25	74.63% (2648/3548)	88.29% (294/333)	0.39 (0.27–0.55)	< 0.00001*

*Statistical significance; *OR* odds ratio, *CI* confidence interval

#### Time-dependent frequency of complete surgical staging of FIGO stage I-IIIA ovarian cancer

Annual changes in the frequency of complete surgical staging of FIGO stage I-IIIA ovarian cancer depending on the number of treated cases were investigated. In centers with lower numbers of treated ovarian cancers the frequency of complete surgical staging decreased between 2020 and 2022 from 76.92 to 72.24% (threshold < 15, Table 3).

**Table 3:** Complete surgical staging of patients with FIGO stage I-IIIA ovarian cancer per year depending on the number of treated ovarian cancer cases per center

Threshold	Year	Frequency of surgical staging in centers < threshold	Frequency of surgical staging in centers ≥ threshold	OR (95% CI)	*p*-value
< 10	2020	78.90% (546/692)	74.21% (449/605)	1.30 (1.00-1.70)	0.05404
	2021	72.94% (477/654)	81.54% (499/612)	0.61 (0.46–0.80)	0.00035*
	2022	74.02% (490/622)	73.32% (481/656)	1.04 (0.80–1.33)	0.82284
< 15	2020	76.92% (710/923)	76.20% (285/374)	1.04 (0.77–1.39)	0.83729
	2021	75.86% (748/986)	81.43% (228/280)	0.72 (0.50–1.01)	0.06072
	2022	72.24% (734/1016)	78.48% (237/302)	0.71 (0.52–0.98)	0.03708*
< 20	2020	75.52% (867/1148)	85.91% (128/149)	0.51 (0.30–0.83)	0.00656*
	2021	76.08% (881/1158)	87.96% (95/108)	0.44 (0.22–0.80)	0.00713*
	2022	72.73% (872/1199)	83.19% (99/119)	0.54 (0.31–0.90)	0.01811*
< 25	2020	75.51% (882/1168)	87.60% (113/129)	0.44 (0.24–0.76)	0.00296*
	2021	76.08% (881/1158)	87.96% (95/108)	0.44 (0.22–0.80)	0.00713*
	2022	72.42% (885/1222)	89.58% (86/96)	0.31 (0.14–0.60)	0.00038*

*Statistical significance; *OR* odds ratio, *CI* confidence interval

#### Association of frequency of complete surgical staging of FIGO stage I-IIIA ovarian cancer with teaching status

Surgical staging was performed less frequently in non-teaching centers compared to university centers (55.45% versus 78.29%: OR 0.35, 95% CI 0.26–0.46, *p* < 0.0001; Table 4).

**Table 4:** Complete surgical staging of patients with FIGO stage I-IIIA ovarian cancer depending on teaching status

Center characteristics			
Teaching status	Non-teaching center (*N* = 15 centers)	Non-university teaching center (*N* = 132 centers)	University center (*N* = 46 centers)
Frequency of surgical staging	55.45% (122/220)	76.30% (1774/2325)	78.29% (1046/1336)
OR (95% CI)	0.35 (0.26–0.46)	0.89 (0.76–1.05)	reference group
p-value	< 0.00001*	0.16783	

*Statistical significance, *OR* odds ratio, *CI* confidence interval

### Macroscopic complete resection of FIGO stage IIB-IV ovarian cancer

#### Frequency of macroscopic complete resection of FIGO stage IIB-IV ovarian cancer depending on the number of treated cases

7,219 patients with FIGO stage IIB-IV ovarian cancer treated between 2020 and 2022 were included in the analysis. Macroscopic complete resection was achieved between 72.3% and 75.6% of cases in all four threshold levels (Table 5). Higher volume centers achieved macroscopic complete resection by about 2 percentage points more frequently. This small difference did not reach statistical significance when using the thresholds 15, 20 and 25. Centers treating less than 10 ovarian cancer cases per year reached macroscopic complete resection less frequently than centers treating 10 patients or more (OR 0.87; 95% CI 0.76–0.99; *p* = 0.03086, Table 5). However, the difference was only about 3 percentage points.

**Table 5:** Macroscopic complete resection during surgery of FIGO stage IIB-IV ovarian cancer

Threshold	Frequency of macroscopic complete resection in centers < threshold	Frequency of macroscopic complete resection in centers ≥ threshold	OR (95% CI)	*p*-value
< 10	72.32% (1066/1474)	75.11% (4315/5745)	0.87 (0.76–0.99)	0.03086*
< 15	73.51% (2436/3314)	75.42% (2945/3905)	0.90 (0.81–1.01)	0.06740
< 20	73.71% (2995/4063)	75.6% (2386/3156)	0.91 (0.81–1.01)	0.07197
< 25	74.04% (3664/4949)	75.64% (1717/2270)	0.92 (0.82–1.03)	0.15473

*Statistical significance; *OR* odds ratio, *CI* confidence interval

#### Time-dependent frequency of macroscopic complete resection of FIGO stage IIB-IV ovarian cancer depending on the number of treated cases

In a next step, annual changes in the frequency of macroscopic complete resection of FIGO stage IIB-IV ovarian cancer depending on the number of treated FIGO stage IIB-IV ovarian cancer cases were investigated. In 2020, frequency of complete macroscopic resection was lower in centers treating lower numbers of cases (72% vs. 77.9%; < 15 vs. ≥ 15 cases, *p* = 0.00138, Table 6). However, in the years 2021 and 2022 this difference was no longer apparent and the frequency of macroscopic complete resection during surgery was comparable for the thresholds 15, 20 and 25 (in 2022: 74.2% vs. 75.9%; <15 vs. ≥ 15 cases, Table 6). The comparison between centers performing fewer than 10 ovarian cancer surgeries per year and those treating at least 10 cases annually showed higher rates of macroscopic complete resection among the higher-volume centers in 2022 (71.4% versus 76.3%; OR 0.78; 95% CI 0.63–0.94; *p* = 0.0208). In the years 2020 and 2021 no difference was observed (Table 6).

**Table 6:** Macroscopic complete resection during surgery of FIGO stage IIB-IV ovarian cancer per year depending on the number of treated ovarian cancer cases per center

Threshold	Year	Frequency of macroscopic complete resection in centers < threshold	Frequency of macroscopic complete resection in centers ≥ threshold	OR (95% CI)	*p*-value
< 10	2020	71.40% (307/430)	75.99% (1440/1895)	0.79 (0.62–1.01)	0.05384
	2021	74.27% (355/478)	73.00% (1363/1867)	1.07 (0.84–1.35)	0.61794
	2022	71.38% (404/566)	76.25% (1512/1983)	0.78 (0.63–0.94)	0.02088*
< 15	2020	72.07% (792/1099)	77.90% (955/1226)	0.73 (0.60–0.89)	0.00138*
	2021	74.29% (858/1155)	72.27% (860/1190)	1.11 (0.92–1.34)	0.29072
	2022	74.15% (786/1060)	75.89% (1130/1489)	0.91 (0.76–1.10)	0.33957
< 20	2020	73.28% (979/1336)	77.65% (768/989)	0.79 (0.65–0.96)	0.01803*
	2021	73.09% (1005/1375)	73.51% (713/970)	0.98 (0.81–1.18)	0.86042
	2022	74.78% (1011/1352)	75.61% (905/1197)	0.96 (0.80–1.15)	0.66232
< 25	2020	73.88% (1182/1600)	77.93% (565/725)	0.80 (0.65–0.99)	0.04091*
	2021	73.17% (1227/1677)	73.50% (491/668)	0.98 (0.80–1.21)	0.90880
	2022	75.06% (1255/1672)	75.37% (661/877)	0.98 (0.81–1.19)	0.90110

*Statistical significance; *OR* odds ratio, *CI* confidence interval

#### Frequency of macroscopic complete resection of FIGO stage IIB-IV ovarian cancer depending on teaching status

We did not observe a difference in the rate of macroscopic complete resection of FIGO stage IIB-IV ovarian cancers between university centres, non-university teaching centres and non-teaching centres (Table 7).

**Table 7:** Macroscopic complete resection during surgery of FIGO stage IIB-IV ovarian cancer depending on teaching status

Center characteristics			
Teaching status	Non-teaching center (15 centers)	Non-university teaching center (132 centers)	University center (46 centers)
Frequency of macroscopic complete resection	77.62% (274/353)	74.41% (3094/4158)	74.34% (2013/2708)
OR (95% CI) p-value	1.20 (0.92–1.57)	1.00 (0.90–1.21)	Reference group
	0.18213	0.94418	

*Statistical significance; *OR* odds ratio, *CI* confidence interval

## Discussion

In many national cancer control plans health authorities ask for minimal treatment volumes with the intention to improve the quality of cancer care. However, there is no convincing evidence that treatment volume is the main factor to determine quality of care and outcome. In this study, which analyzed data from nearly 4,000 FIGO stage I-IIIA - ovarian cancer patients, we observed that an annual treatment volume of 15 ovarian cancer cases and more improved the rate of optimal surgical staging of FIGO stage I-IIIA ovarian cancer (*n* < 15 vs. *n* ≥ 15: 74.9% vs. 78.5%, *p* = 0.03096). This difference increased when the threshold of treated ovarian cancer cases was 20 and 25, respectively (*p* < 0.00001). In contrast to this finding, it was shown that in certified gynecological cancer centers the rate of macroscopic complete resection in about 7,000 patients with FIGO IIB-IV ovarian cancer is not associated with surgical volume except in the lowest threshold analysis. Based on the available data, it was not possible to distinguish between patients who underwent primary cytoreductive surgery and those who received neoadjuvant chemotherapy followed by surgical treatment. In our analysis using certification data of gynecological cancer centers with an annual number of treated ovarian cancer cases of less than 15, the rate of macroscopic complete resection was 73.5% compared to 75.4% in centers with treatment volumes of 15 cases or more per year (*p* = 0.0674). With rising thresholds for treatment numbers this statistically insignificant difference became even smaller. Although we assumed that treatment volume would lead to increased rates of macroscopic complete resection during surgery because of better surgical expertise in high volume centers this was not the case. In contrast, it was not expected that rates of optimal surgical staging of FIGO I-IIIA ovarian cancer that includes procedures that need less surgical expertise were achieved more often in centers with higher volume especially in university hospitals. A recent study by Wimberger et al. demonstrated that optimal surgical staging quality (defined as ≤ 1 missing staging procedure) of patients with early ovarian cancer FIGO stage I increased over time from 19.9 to 54.1% between 2004 and 2016 in a retrospective study based on questionnaires sent to all German hospitals with higher increases in university hospitals (Wimberger et al. 2023). Suboptimal surgical staging led to reduced survival by about 21% after 48 months. Both studies indicate that expertise, education and certified interdisciplinary cancer center structures may be more important than treatment volume for high quality of care.

Several previous studies that included mainly patients with advanced ovarian cancer, indicated that surgical volume is associated with improved survival (Kumpulainen et al. 2002; Bristow et al. 2010; Cliby et al. 2015; Wright et al. 2019; Moterani et al. 2020; Prost et al. 2024). However, it is unclear whether surgical volume is the main factor which determines survival. In their retrospective population-based study Bristow et al. included 9,933 ovarian cancer patients between January 1, 1996 and December 31, 2006 (Bristow et al. 2015). 2,269 (22.8%) of the included patients had stage I, 782 (7.9%) stage II, 4,479 (45.1%) stage III and 2,402 (24.2%) stage IV ovarian cancer. They showed that ovarian cancer-specific survival was significantly better for patients treated in National Cancer Institute-Designated Comprehensive Cancer Centers (NCI-CCC) compared to high-volume hospitals, with both having an average annual case volume of 14.5 and 14.6 per year, respectively (ovarian cancer-specific survival: 77.9 months, vs. 51.9 months, *p* < 0.0001) (Bristow et al. 2015). The impact of guideline adherence along with the surgical volume on survival of ovarian cancer patients was investigated by Cliby et al. in their retrospective cohort study including 96,802 ovarian cancer cases between 1998 and 2007 (Cliby et al. 2015). Data was obtained by the National Cancer Data Base capturing 80% of all US cases. 19,516 (17.43%) of the included patients had stage I, 7,941 (7.09%) stage II, 43,918 (39.23%) stage III and 27,587 (24.64%) stage IV ovarian cancer (unknown/unclear stage in 12,994 patients (11,3%). Delivery of non-guideline care (OR 1.4, 95% CI 1.36–1.44), and higher facility case volume (OR 0.91, 95% CI 0.86–0.96) were both independent predictors of overall survival. With an adjusted HR for overall survival of 0.920 (95% CI 0.876-0.99695) when comparing treatment in hospitals with a volume of 15–25 vs.1–6 cases/year the effect of higher volume of treated patients was small but significant. High volume centers were less likely to deliver non-guideline care (OR 0.44, 95% CI 0.41–0.47) with guideline adherence being an independent predictor of overall survival (adjusted HR for overall survival adherence to NCCN guidelines: no vs. yes: HR 1.403; 95% CI 1.362–1.446) (Cliby et al. 2015).

The question of whether hospital volume or experience of physicians improves survival, was investigated in the retrospective study of Bristow et al. in 2014 (Bristow et al. 2014). 11,865 ovarian cancer patients with stage IIC-IV disease included between January 1, 1996 and December 31, 2006. Data was obtained from the California Cancer Registry. Institutions were defined as high-volume hospitals (HVH) when they treated ≥ 20 cases/year and physicians were referred to high-volume physician (HVP) when treating ≥ 10 cases/year. They showed that treatment in a HVH by a HVP was associated with a significantly higher ovarian cancer-specific survival compared to the combination low-volume hospital (LVH)/low-volume physician (LVP) (HR 1.31; 95% CI 1.16–1.49). When either experience of the physician or hospital volume was low, survival benefit was no longer significant (HVH/HVP vs. HVH/LVP: HR 1.14; 95% CI 0.98–1.31 and HVH/HVP vs. LVH/HVP: HR 1.08; 95% CI 0.94–1.25) (Bristow et al. 2014). This issue was also investigated by Vernooij et al. in a retrospective cohort study, including 1,077 ovarian cancer patients treated between 1996 and 2003 in The Netherlands (Vernooij et al. 2009). They observed that the level of specialization, volume of hospitals and expertise of gynecologists was strongly related to the proportion of adequately staged patients (adjusted OR specialized hospitals 3.9 (95% CI 2.0–7.6); specialized gynaecologists 9.5 (95% CI 4.7–19). Patients with stage III disease had a higher chance of optimal debulking when treated in specialized hospitals (adjusted OR 1.7, 95% CI 1.1–2.7) or by high volume gynaecologists (adjusted OR 2.8, 95% CI 1.4–5.7). Overall survival was best for patients treated in specialized hospitals and by high-volume gynecologists. In this study, ovarian cancer patient volume of the hospital did not have a significant influence on survival. Taken together, this is another study, emphasizing the beneficial role of hospital specialization and expertise of the treating physicians determining patient outcome.

Although there is some evidence for the benefit of volume on long-term survival, some studies have shown increased short-term mortality of patients treated in high-volume centers. The “Dutch Gynecological Oncology Audit Collaborator Group” recently investigated the effect of surgical volume on short-term outcomes of macroscopic complete resection for advanced stage ovarian cancer (Algera et al. 2024). In previous studies they described that centralization of care increased the Dutch benchmark for the complete result of cytoreductive surgery (no macroscopic residual disease rose from < 50% in 2008 to up to 70% in 2017–2020) (Timmermans et al. 2019; Baldewpersad Tewarie et al. 2021; Algera et al. 2023). The surgical volume of these centers was at least 20 cases. In the recent study five high volume centers (54–84 cases) achieved improved macroscopic complete resection rates compared to institutions with lower case numbers (OR 1.9; 95% CI 1.2–3.1). However, these were associated with increased severe complication rates (OR 2.3;95% CI 1.3–4.2) in the group of primary cytoreductive surgery. The authors argued that these results do not support the intention to further centralize ovarian cancer care as proposed by Dutch health authorities (case volume 50 per center).

In line with this, in the retrospective study including 16,089 women treated for ovarian cancer performed by Shakeel et al. volume effect regarding in-hospital mortality was small (OR 0.95; 95% CI 0.91 to 0.99) (Shakeel et al. 2017). However, they observed, that if surgery was performed by high-volume surgeons, the risk of major complications was significantly higher (OR 1.12; 95% CI 1.02 to 1.23). Underlying reason for these increased complication rates could be that on the one hand hospitals with high case numbers and a high level of expertise perform more complicated surgical treatments, and on the other hand, they might perform more radical surgery leading to more complications.

Overall, these data suggest that higher volume of ovarian cancer surgeries may be associated with improvement of long-term survival of patients with ovarian cancer. However, current evidence suggests that other factors of quality in specialized cancer centers may be more important than treatment volume alone. Quality of cancer care can be improved by the establishment of certification programs.

One possible reason for improved survival of patients treated in gynecological cancer centers or high-volume hospitals compared to those treated in non-certified or low-volume institutions is the higher rate of macroscopic complete resection during surgeries (Kumpulainen et al. 2009; Dahm-Kähler et al. 2016). In their combined exploratory analysis of 3 prospecively randomized phase 3 multicenter trials, du Bois et al. included data of 3,126 patients and observed significantly better overall survival and progression-free survival of ovarian cancer patients after macroscopic complete resection during surgery compared to those with residual disease between 1 and 10 mm (multivariable analysis: HR 2.12; 95% CI 1.85–2.43; *p* < 0.0001) (Du Bois et al. 2009). Consistent with this, the German Quality Management Program observed 5-year survival rates of 55% in patients with complete resection compared to 16% in women with a residual tumor of 1–10 mm and 13% in patients with a residual > 1 cm (*p* < 0.001) (Harter et al. 2011). Chi et al. reported in 2009 that more extensive debulking of upper abdominal disease in patients with advanced ovarian cancer was associated with significantly better progression-free and overall survival (progression-free survival: HR 0.757; 95% CI 0.601–0.953; *p* = 0.01; overall survival: HR 0.764; 95% CI 0.592–0.987; *p* = 0.03) (Chi et al. 2009). In conclusion, these data suggest that macroscopic complete resection during surgery improves survival of ovarian cancer patients.

In Germany, the certification of oncological treatment facilities focusses on the implementation of evidence-based treatment guidelines and verifies adherence to quality indicators (Schmitt et al. 2023). The study “Effectiveness of Care in Certified Cancer Centers (WiZen)” included 11 cancer entities to investigate whether initial treatment in hospitals with or without certification was associated with a difference in overall survival (Schmitt et al. 2023). In that study, overall survival of ovarian cancer patients that were treated in certified cancer centers was shown to be significantly longer compared to patients treated in non-certified institutions (HR 0.88; 95% CI 0.82–0.95; *p* = 0.001) (Schmitt et al. 2023).

In early ovarian cancer, incomplete staging has already been identified as a common problem in several studies (McGowan et al. 1985; Zanetta et al. 1998; Sijmons et al. 2007; Timmers et al. 2010; Hengeveld et al. 2019; Laven et al. 2022). In 2007, Sijmons et al. analyzing data from the Dutch regional cancer registry between 1991 and 1997 found that only 41 of 125 patients (32.8%) with early ovarian cancer received optimal staging (Sijmons et al. 2007). Those who received optimal staging had a significantly lower risk of death than those with incomplete staging (HR 7.4; 95% CI 1.7–32.2) (Sijmons et al. 2007). The 5-year survival was 97.6% for patients with optimal staging and 68.5% without the corresponding diagnostic steps (Sijmons et al. 2007). Similarly, in another Italian retrospective study, completeness of staging as identified as an independent prognostic factor for progression-free survival and overall survival (*p* = 0.007 and *p* = 0.0008, respectively), with the strongest impact seen in poorly differentiated tumors (Zanetta et al. 1998). Similar results were shown more recently in a retrospective study by Hengeveld et al. (2019). Data from patients with stage I ovarian cancer from all over Denmark and a Dutch hospital between 2005 and 2017 were analyzed (Hengeveld et al. 2019). The collective comprised 1,234 women, 65.5% of whom had neither individual lymph nodes resected nor a systematic LNE performed. Furthermore, 31.8% of cases were upstaged from apparent stage I, but only 4.7% were upstaged due to metastatic lymph nodes. Of these 393 upstaged patients, 35.1% were reated differently due to the surgical findings (Hengeveld et al. 2019). The problem of incomplete staging was also observed in 66% of patients in the randomized Adjuvant Chemotherapy In varian Neoplasm (ACTION) study of the European Organisation for Research and Treatment of Cancer (EORTC), although comprehensive surgical staging was explicitly recommended in the study protocol (Timmers et al. 2010).

The strengths of our study are the high number of included cases and the reliability of data obtained from certified cancer centers, and data completeness. Moreover, the number of cases in the subgroups especially for the analysis of macroscopic complete resection as a strong surrogate parameter for survival, but also for the investigation of FIGO stage I-IIIA ovarian cancer is well balanced, which increases statistical power.

However, there are also some limitations of our study. As the underlying data was provided by the certification program, available information was limited to center-aggregated data. Thus, correlation of our data with survival of ovarian cancer patients was not possible. Moreover, the rate of macroscopic complete resection during primary surgical treatment compared to that after neoadjuvant chemotherapy cannot be determined which may bias the present results. However, the German S3-guideline “diagnosis, treatment and follow-up of malignant tumors of the ovary“ clearly recommends primary surgical treatment of ovarian cancer (S3-Guideline “Diagnosis, Treatment and Follow-up of malignant Ovarian Tumors”). Therefore, it can be expected that the number of neoadjuvant treated cases is relatively low. Another limitation is that the quality indicator “macroscopic complete resection” is based on a subjective judgement. Nevertheless, as described above, several previous studies that were also based on self-reporting showed that achieving macroscopic complete resection still leads to a survival benefit for ovarian cancer patients supporting its relevance for evaluation of ovarian cancer care.

## Conclusion

We observed in our study that a rate of 75% of macroscopic complete resection during surgery of patients with FIGO stage IIB-IV ovarian cancer was achieved in certified gynecological cancer centers. Except when looking at the < 10 threshold, this was not associated with treatment volume in our study sample. In contrast, optimal staging during surgery of patients with FIGO I-IIIA ovarian cancer was higher in certified hospitals treating 15 ovarian cancer patients or more each year. Both, macroscopic complete resection in FIGO stage IIB-IV ovarian cancer and optimal surgical staging in FIGO stage I-IIIA ovarian cancer are known to be associated with improved survival of the patients. These data contribute to future discussions regarding improvement of ovarian cancer care.

## Data Availability

The datasets generated during and/or analyzed during the current study are available from the corresponding author on reasonable request.
